# Trauma: the role of the innate immune system

**DOI:** 10.1186/1749-7922-1-15

**Published:** 2006-05-20

**Authors:** F Hietbrink, L Koenderman, GT Rijkers, LPH Leenen

**Affiliations:** 1 Dept. of Surgery, University Medical Center Utrecht, The Netherlands; 2 Dept. of Pulmonary Science, University Medical Center Utrecht, The Netherlands; 3 Dept. of Immunology, University Medical Center Utrecht, The Netherlands

## Abstract

Immune dysfunction can provoke (multiple) organ failure in severely injured patients. This dysfunction manifests in two forms, which follow a biphasic pattern. During the first phase, in addition to the injury by trauma, organ damage is caused by the immune system during a systemic inflammatory response. During the second phase the patient is more susceptible for sepsis due to host defence failure (immune paralysis). The pathophysiological model outlined in this review encompasses etiological factors and the contribution of the innate immune system in the end organ damage. The etiological factors can be divided into intrinsic (genetic predisposition and physiological status) and extrinsic components (type of injury or "traumaload" and surgery or "intervention load"). Of all the factors, the intervention load is the only one which, can be altered by the attending emergency physician. Adjustment of the therapeutic approach and choice of the most appropriate treatment strategy can minimize the damage caused by the immune response and prevent the development of immunological paralysis. This review provides a pathophysiological basis for the damage control concept, in which a staged approach of surgery and post-traumatic immunomonitoring have become important aspects of the treatment protocol. The innate immune system is the main objective of immunomonitoring as it has the most prominent role in organ failure after trauma. Polymorphonuclear phagocytes and monocytes are the main effector-cells of the innate immune system in the processes that lead to organ failure. These cells are controlled by cytokines, chemokines, complement factors and specific tissue signals. The contribution of tissue barrier integrity and its interaction with the innate immune system is further evaluated.

## Introduction

Trauma is one of the major causes of mortality in people under the age of 50 in the Western world. Patients die as a direct consequence of their sustained injuries, or by the additional damage caused by subsequent immune reactions [1]. About 5% of the patients admitted after severe trauma develops (multiple) organ failure (MOF). Multiple organ failure is a clinical syndrome in which the functionality of several organs fail subsequently or simultaneously (i.e. liver, lungs, kidneys, heart). This review outlines the initiating factors and underlying mechanisms for the development of post-traumatic organ failure. It provides a pathophysiological basis for the so-called damage control concept. This concept involves a treatment strategy in which a staged approach of surgery in severely injured patients and post-traumatic immunomonitoring have become important aspects, to minimize the negative effects of a dysfunctional innate immune system.

## Multiple organ failure

**Figure 1:**
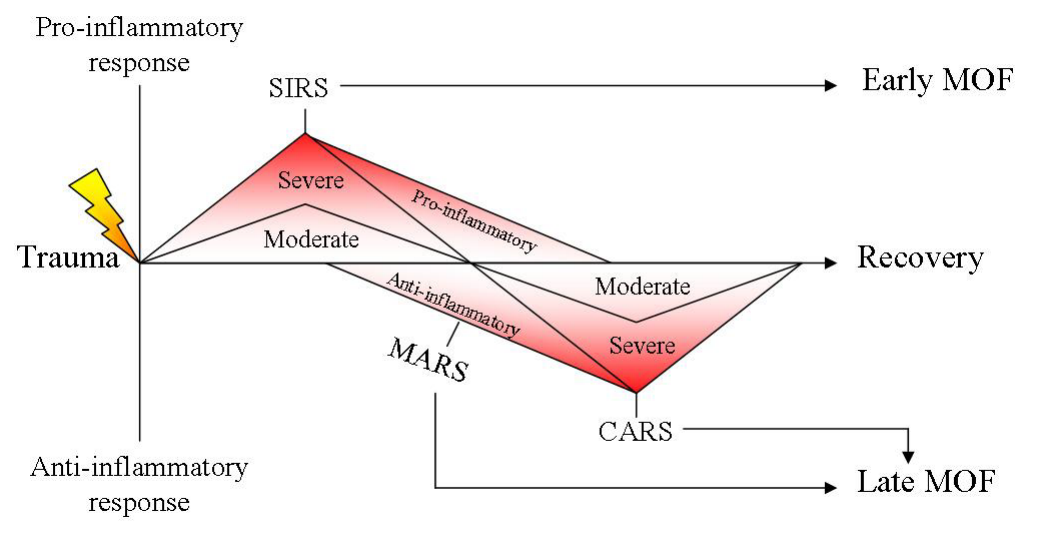
**Biphasic model of organ failure**. Depiction of the biphasic model of organ failure (MOF), originally coined by Moore[8]. The relative degree of immune activation is displayed on an arbitrary scale on the vertical axis. The horizontal axis indicates the time following trauma. When injury is sustained, a systemic pro-inflammatory response (SIRS) is evoked which can lead to the early version of MOF. At a later stage a compensatory anti-inflammatory response syndrome (CARS) or mixed antagonist response syndrome (MARS) can lead to immune paralysis and subsequently, the late form of organ failure.

Multiple organ failure after trauma has a multifactorial etiology, which can be divided in endogenous and exogenous factors. Endogenous factors, such as genetic predisposition and physical condition form the basis of the patient s susceptibility for the development of organ failure. Recent studies have shown that genetic variations (e.g. TNF-α polymorphisms) are strongly associated with the development of organ failure [2]. Exogenous factors, like the injury itself (the "first hit" or "trauma-load") and the resuscitation or surgical intervention (the "second hit" or "intervention load") play a key role in the development and clinical presentation of organ failure. Organ damage and subsequent organ failure is the result of a dysfunctional immune system. A localized inflammatory reaction after injury is physiological, which can be explained by the "danger model", an immunological theory coined by Matzinger. The "danger model" explains that alarm signals can provoke an inflammatory reaction [3]. These alarm signals can be secreted by healthy cells or released by necrotic cells, which are present after injury is sustained. The combination of type of tissue and type of alarm signal decides what kind of response follows. Neutrophils and macrophages (effectors) are involved in immune surveillance and injury control and after trauma are activated through mediators (cytokines, chemokines and complement). This local inflammatory response can exacerbate and a systemic inflammatory response (SIRS) develops. When SIRS leads to a multiple organ dysfunction syndrome (MODS) mortality can increase up to 50–80% (Fig. 1) [2,4,5].

**Figure 2:**
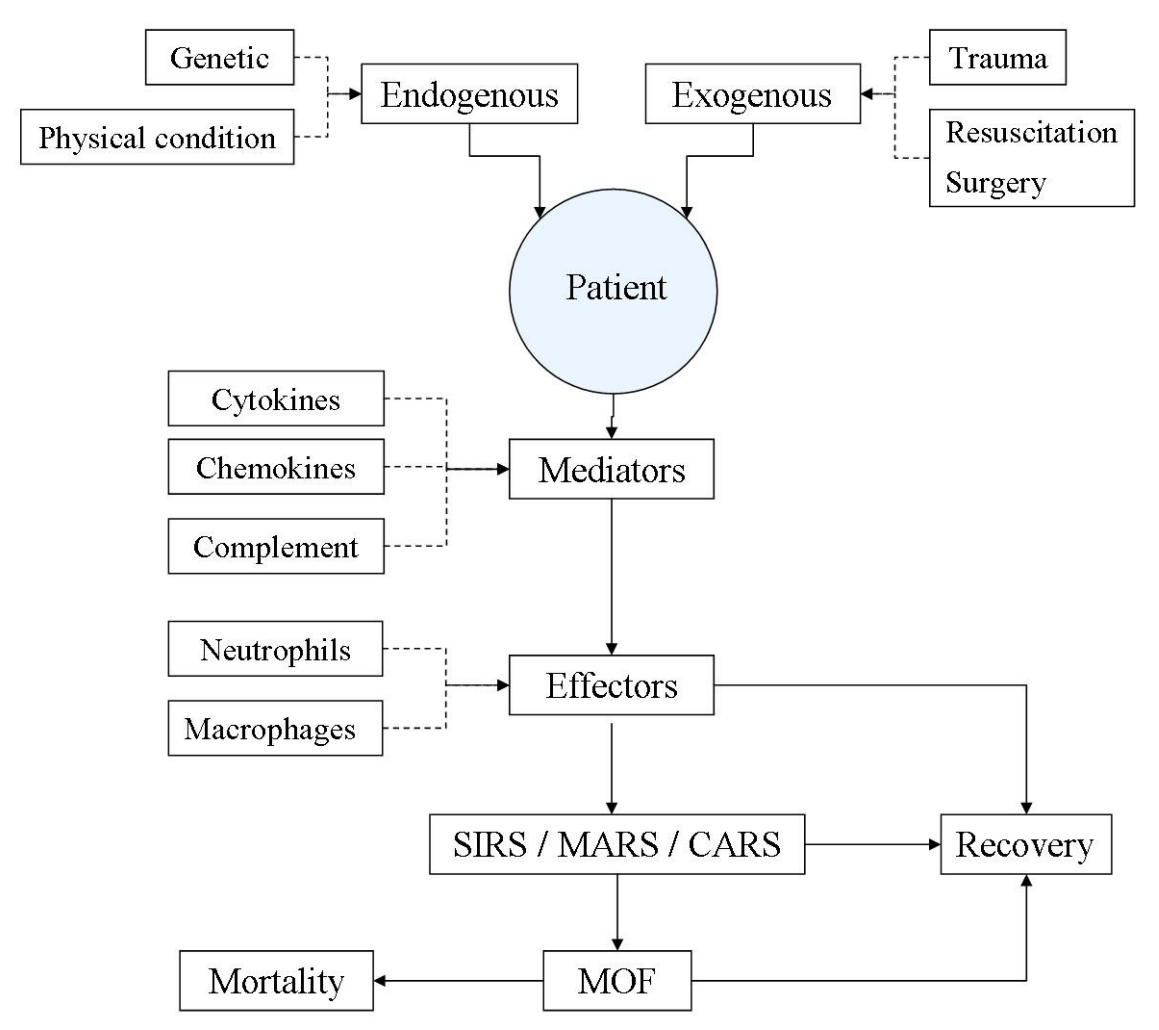
**Factors involved in the etiology of post-traumaticorgan failure**. Shows the complex of factors, mediators and effectors involved in the development of organ failure. The endogenic factors (genetic predisposition and physical condition) form the basis for the susceptibility of a patient to post-traumatic organ failure. The sustained injury is seen as the first hit on the immune response and the "burden of surgery" is seen as the second hit, which can excacerbate the inflammatory reaction. The mediators stimulate the effectors which cause end-organ damage.

To restore the equilibrium of the excessive pro-inflammatory reaction, an anti-inflammatory response is evoked. In a propitious case, homeostasis is achieved. However, an overreaction of the anti-inflammatory response can lead to either a compensatory anti-inflammatory response (CARS), or a mixed antagonist response (MARS) [6]. In the latter syndrome the pro-inflammatory and anti-inflammatory responses counterbalance each other. In both situations (CARS and MARS), the body is in a state of immune paralysis and is unable to produce an adequate reaction to a new threat (i.e. infection). In this state the patient is extremely prone to micro-organisms as there is a defect in an important defense mechanism formed by the cells of the innate immune system [7]. Resulting infections can cause serious complications like sepsis and septic shock with subsequent organ failure [8]. In conclusion, SIRS and sepsis (predisposed by CARS or MARS), despite different pathophysiological processes, can all result in multiple organ failure (Fig. 2).

## Cellular response: neutrophils

**Figure 3:**
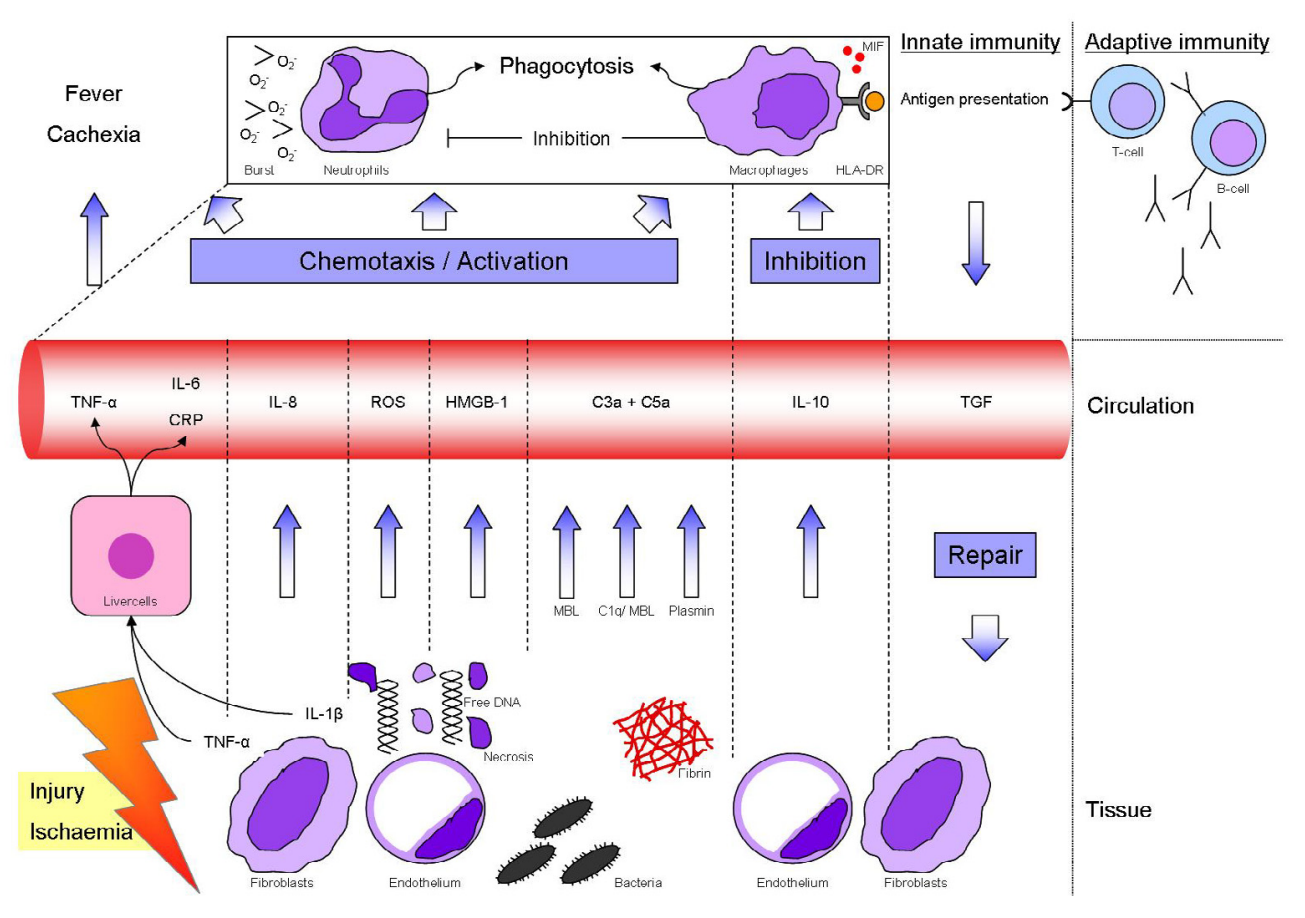
**Innate immunity in tissue damage**. Shows the relation between several important factors involved in the pathophysiology of organ failure after tissue injury. The figure is explained in detail in the article. C3a: Complement factor 3a; C5a: Complement factor 5a; O_2_^-^: Radical oxygen; MBL: Mannose binding lectin; C1q: Complement factor 1q

Tissue damage leads to the activation of neutrophils and macrophages [9]. Hemorrhagic shock induces ischemia and this causes the tissue to change its metabolism to anaerobic. During resuscitation, thus reperfusion, oxygen is transported to the ischemic area in the tissue and radical oxygen species (ROS) are formed. These ROS are chemo-attractants and activators of neutrophils (Fig. 3) [10,11]. Polymorphonuclear granulocytes (PMNs) have an important role in the defense and debridement of the injured tissue from the first 10 minutes until 3 days after injury [12]. Priming, or pre-activation, is an essential step for neutrophils which enhances functional responses of these cells [13,14].

### Priming

Priming is the result of pre-exposure to priming agents, like granulocyte macrophage colony stimulating factor (GM-CSF) or tumor necrosis factor (TNF-α) [15,16]. These priming agents are found in increased concentrations in the peripheral blood of severely injured patients and several priming enhanced functions of neutrophils have been demonstrated in traumapatients and patients undergoing major abdominal surgery [17,18]. The enhanced functional response after priming encompasses chemotaxis, adhesion, rolling, diapedesis and the oxidative burst.

### Oxidative burst

The increased oxidative burst (a cytotoxicity associated response) is necessary to prepare the neutrophils for invading micro-organisms. This increased functional response in the form of oxidative radical production correlates with the incidence of SIRS and MOF [19]. It is thought that the increased cytotoxic potential of neutrophils is a sign of an uncontrolled inflammatory reaction, which causes damage to tissues and leads to early MOF. Maximum increased priming for cytotixicity (after *in vitro *stimulation) was found between 3 and 24 hours after trauma [20]. An elevated priming index (elevation of the spontaneous oxidative burst from normal values) was found between day 2 and 5 after trauma and remained above normal until day 13 after trauma [21]. This increased oxidative burst is thought to cause additional damage to the tissue. Furthermore, the newly formed ROS contribute to the attraction and subsequent activation of neutrophils, which attributes to the accumulation of activated neutrophils in the tissue [11]. The harmful effects of neutrophil activity can only occur when these cells enter the tissue, therefore, an interaction between the neutrophil and endothelium has to occur. Interactive processes with the endothelium, like rolling, adhesion and diapedesis, are necessary for leukocytes to exert their function in the target tissue. These leukocyte functions are altered after trauma and during early organ failure.

### Rolling

Rolling is regulated and controlled by selectins. These proteins undergo interactions with ligands on endothelial cells, which slow down the leukocytes at this surface [22]. E-selectin, which can bind carbohydrate molecules, is presented on endothelial cells and are involved in the initial contact between endothelial cells and leukocytes. Leukocytes express L-selectin on their surface and is important in secondary tattering, a process in which attached leukocytes provide adhesion for other leukocytes. As a result, leukocytes bind directly to each other and thus enhance the effect of the homing process [23]. L-selectin is shed after interaction with the endothelium and integrins take over to regulate the next step in the transmigration process. Some authors have reported a correlation between decreased L-selectin expression on leukocytes and the incidence of SIRS or early MOF, indicating to a relation between the degree of neutrophil activation and the development of complications occurring during the pro-inflammatory phase [24,25]. The shed molecules can be found as soluble factors in serum (sL-selectin). Consequently, the activation level of the neutrophil population is associated with the level of sL-selectin in the blood. Maximum sL-selectin levels in serum are found 6 hours after trauma, giving an indication on the time when the highest amount of neutrophils have lost their L-selectin to migrate to the tissue [26].

### Adhesion

Integrins are involved in the adhesion of leukocytes to the endothelium. The integrin amβ2, or MAC-1 (CD11b/CD18) and the ligand ICAM-1 (intercellular adhesion molecule 1) form a high affinity stationary connection between leukocyte and endothelium. This is in contrast to the low affinity, reversible binding of selectins. Functional integrins are only expressed upon activation of the neutrophil and are necessary for an adequate transmigration process [27]. An increased expression of MAC-1 is found on neutrophils from patients who were admitted with an ISS > 16 as compared to traumapatients with an ISS < 16, indicating to activated neutrophils after injury [26]. Increased expression of MAC-1 is also found in experimental models and patients who received large amounts of blood products for resuscitation [28]. In contrast, during late organ failure a decreased expression of MAC-1 is found on neutrophils from patients who died from the consequences of sepsis as compared to patients who survived [29]. These results are congruent with the decreased percentage of MAC-1 positive neutrophils of critically ill surgical patients with severe disease as compared with surgical intensive care patients with less severe disease [30].

ICAM-1, normally expressed by activated entothelium, also exists as a soluble factor in serum (sICAM-1) and increased concentrations in septic patients correlate with the incidence of organ failure and mortality [26,29]. Expression of MAC-1 or sICAM give an indication on the activation of neutrophils or tissue and are both related with the development of organ failure. A high activation state of neutrophils is associated with SIRS, whereas a low activation state is related with sepsis. The activation state of neutrophils changes over time and could provide a partial explanation for the biphasic pattern of MOF [8].

### Apoptosis

Billions of neutrophils are produced by the bone marrow on a daily basis [31]. Neutrophils, which have completed their function in the tissue, go into apoptosis. Apoptosis is necessary to limit the absolute number of neutrophils present in the tissues. After trauma a delayed programmed cell death (delayed apoptosis), has been demonstrated [21]. This delay is seen directly after trauma and can last up to 3 weeks [32]. Delayed apoptosis causes accumulation of neutrophils in the tissue, where they can produce more cytotoxic products (oxygen radicals and proteases) and promote tissue damage. This delayed apoptosis is found in patients with sepsis as well [33]. Bacterial products can inhibit apoptosis. In contrast to the large population of neutrophils which show decreased apoptosis, a relative larger subgroup of neutrophils exhibits signs of apoptosis in whole blood [34].

Neutrophils are essential in the pathophysiology of trauma-related organ failure [35]. Blocking or depletion of neutrophils in experimental models results in a reduction of organ failure in the pro-inflammatory (early) phase. However, overall organ failure increased due to an increased incidence of organ failure caused by severe infections during the anti-inflammatory (late) phase [36]. For future studies it seems more favorable to regulate the neutrophil compartment instead of shutting this important defense mechanism down.

## Cellular response: macrophages

Neutrophils are important in the first response to injury, as they form the first natural immunological defense against micro-organisms and occur within 10 minutes after injury is sustained. Subsequent to the initial responders, monocytes/macrophages are recruited. These cells orchestrate the mechanisms involved in wound healing [37]. They function in wound debridement and secrete biologically active substances, called growth factors (e.g. TGF). TGF plays an important role in cell growth and tissue repair and thus essential in the wound repair after trauma [38]. Macrophages have a lasting influence on the subsequent phases of proliferation and tissue differentiation. Most of the macrophages are derived from blood monocytes. Differentiation of monocytes into macrophages and activation of macrophages takes place at the wound site. The cells reach the wound area in great numbers, attracted by chemotactic signals from injured tissue, the cytokines produced by immune cells and the presence of bacterial products. A macrophage can phagocytose micro-organisms and, in addition, is also capable of modulation of the adaptive immune response by mediating antigen presentation to lymphocytes. Antigens are taken up and partially degraded by the macrophage and then presented to a T-lymphocyte for recognition by MHC-II molecules. In injured patients, macrophages form the bridge between innate and adaptive immunity.

Down-regulation of MHC-II expression leads to decreased antigen presentation capacity and therefore higher susceptibility for infectious complications. Several authors have shown MHC-II suppression after trauma, which correlated with the incidence of infectious complications. MHC-II suppression on monocytes and macrophages is considered to be one of the most important features of immune suppression after injury. Some authors have suggested CARS to be defined as less than 30% expression of MHC-II on monocytes [29].

## Cytokines and chemokines

In past years many studies focused on the relation between pro- and anti-inflammatory cytokines and the development of SIRS and CARS. Tissue damage causes the endothelial cells, fibroblasts, lymphocytes and tissue-macrophages to produce these cytokines [39]. At first, pro-inflammatory cytokines, such as TNF-α, GM-CSF, interleukin 1β (IL-1β), IL-6 and IL-8 are produced [40].

### TNF-α and IL-1β

TNF-α and IL-1β are situated at the beginning of the pro-inflammatory cascade (Fig. 3). IL-1β acts primarily locally, but induces a systemic release of TNF-α and IL-6 by stimulation of hepatic cells. IL-1β and TNF-α increase the concentration of neutrophils in the circulation, trigger an increased chemotactic response, decrease the apoptosis ratio, amplify phagocytosis and cause an increased permeability of the endothelium. These actions lead to accumulation of activated inflammatory cells in the tissue [41,42]. IL-1β has been identified as an important cytokine in patients with the acute respiratory distress syndrome (ARDS), a neutrophil mediated disease. Only small amounts of biological active IL-1β are necessary to induce inflammation in the pulmonary compartment [41,43]. TNF-α has a more ambiguous role as its function is depending on the context of the tissue. It participates in an adequate immune response in its physiological role in the circulation. TNF-α depleted or inhibit mice were incapable of handling an infectious threat [44]. In addition, administration of TNF-α reduces mortality in a sepsis model performed on rats [45]. In a clinical situation however, increased serum concentrations of TNF-α correlate with the development of septic shock in trauma patients. It is unclear whether this is a causal relationship, or whether this is merely an epiphenomenon and the high levels of TNF-α are a sign of the host coping with tissue injury or invading micro-organisms [46].

### IL-6 and IL-8

Both IL-1β and TNF-α stimulate the production of IL-6 and IL-8. IL-8 is an important chemokine in the cascade that leads to leukocyte recruitment and activation in the tissues [47]. Production of IL-8 induces an influx of neutrophils towards the site of production, for example in patients with ARDS to the lung. The IL-8 concentration in the pulmonary fluid of patients with a thoracic trauma is seen as an indicator for the occurrence of ARDS, as increased levels correlate with the incidence [48]. IL-6 is an acute phase protein such as C-reactive protein (CRP). The protein's role in the pathophysiology of trauma-related organ failure remains unclear due to the non-specificity of IL-6. However, epidemiological data shows evidence of a correlation between increased IL-6 levels after trauma and the Injury Severity Score (ISS), the incidence of complications and mortality. A correlation also exists between the IL-6 concentrations after intramedullary osteosynthesis and the development of ARDS [49]. IL-6 can be seen as marker for the severity of trauma and, despite its indistinct role in the pathophysiology, can be a resource in triage, diagnosis and prognosis.

### MIF

Macrophage migration inhibitory factor (MIF) is a pleiotropic molecule exerting its functions as an anterior pituitary hormone, a pro-inflammatory cytokine and high activity enzyme. It is produced abundantly by monocytes/macrophages and acts in an autocrine/paracrine manner to up-regulate and sustain the activation responses of diverse cell types [50]. MIF is present in preformed, cytoplasmic pools within the macrophage and is *in vitro *rapidly released to microbial products (both lipopolysaccharide and Gram-positive exotoxins) [51]. This is also seen *in vivo *as high circulating levels of MIF were found in septic and septic shock patients, in contrast to normal levels in non-septic traumapatients [52]. In addition, circulating levels of MIF correlated with positive tests for bacterial cultures [53]. MIF induces vascular hyporeactivity and could be the threshold protein in the occurrence of septic shock.

MIF overrides the anti-inflammatory actions of glucocorticoid and acts via the stimulation of pro-inflammatory cytokines like TNF-α, IL-1β and IL-8 via the NF-κB pathway. MIF prevents apoptosis by reduction of the p53 tumor suppressor gene. Therefore, high concentrations of MIF lead to a sustained pro-inflammatory response and delayed apoptosis of cells of the innate immune system. High concentrations of MIF have been found in the alveolar spaces of patients with ARDS [54]. Those authors suggest that MIF acts as a mediator sustaining the inflammatory response in ARDS and that an anti-MIF strategy may represent a novel therapeutic approach in inflammatory diseases like ARDS.

### HMGB-1

High-mobility group box (HMGB)-1 was originally identified as a nuclear DNA-binding protein that functions as a structural cofactor for proper DNA-transcriptional regulation and gene expression [55]. Recent studies indicate that immune cells can liberate HMGB-1 into the extracellular milieu where it functions as a pro-inflammatory cytokine. HMGB-1 is recognized by cells of the immune system as a necrotic marker to signal tissue damage. It can be passively released by damaged or necrotic cells or actively secreted by macrophages and neutrophils. It is seen as a late mediator as it is secreted by macrophages *in vitro *20 hours after stimulation. Increased levels of HMGB-1 result in the disruption of endothelial barrier functions, leading to vascular leakage and tissue hypoperfusion, similar to that observed in sepsis. *In vivo *increased levels of HMGB-1 are shown in patients with severe sepsis [56]. In experimental studies inhibition of HMGB-1 prevents endotoxin and bacteremia induced multiple organ failure and improves survival [57]. In an experimental model intratracheal administration of recombinant HMGB-1 induces a dose-dependent interstitial and intra-alveolar neutrophil accumulation and lung edema at 8 and 24 hours post-administration[58]. Neutralizing HMGB-1 antibodies have been reported to reduce mortality in experimental models of acute lung injury or ischemia/reperfusion injury [55].

### IL-10

IL-10 plays an important role in the anti-inflammatory response. This protein is produced simultaneously with the pro-inflammatory cytokines, but peaks hours later. One of the functions of IL-10 is the negative feedback on the production of TNF-α, IL-6 and IL-8. The cytokine IL-10 plays a pivotal role in the suppression of monocyte function as it directly decreases MHC-II expression [59]. IL-10 causes the MHC-II molecules on the surface of monocytes and macrophages to be internalized [60]. Increased levels of IL-10 have been shown to correlate with the development of sepsis or adverse outcome during sepsis. However, IL-10 is unable to discern outcome or severity of illness on an individual level. In addition, the biological activity of IL-10 is dependent on the pH and temperature, which is often altered in severely injured or septic patients [61]. It is unclear, whether increased IL-10 levels have a causal relationship with the development of complications, or whether it is a sign of a struggling host.

## Complement factors

Complement is a collection of proteins, which are involved in the protection against micro-organisms. It is one of the most preserved defense mechanisms during the evolution of the immune system. Next to activation by immune complexes complement can bind conserved bacteriological compounds (e.g. bacterial carbohydrates, bacterial antigens) and altered self-products (e.g. free DNA) via mannose binding lectin, ficolins or complement factor C1q [62]. Complement can opsonize bacteria by complement factor C3b, a split product of C3. Opsonisation leads to attraction of leukocytes and enhances phagocytosis of bacteria. In the absence of bacterial or altered self products, the complement system can be activated by a connection with the coagulation system. The coagulation cascade and the complement cascade are connected through plasmin, a product of the trombolytic route that regulates homeostasis in the coagulation. Due to injury large scale activation of the coagulation cascade occurs. In trauma both coagulation factors and tissue damage activate the complement cascade [63]. This leads to neutrophil homing to the tissues and activation on the site of injury. Several studies have shown a correlation between activated complement factors (C3a/C3 ratio and C5a) and mortality after trauma [64]. *In vitro *is shown that C5a regulates two important aspects of neutrophil function; i) adhesion associated processes and ii) cytotoxic associated processes [65]. Complement is one of the most important factors contributing to neutrophil dysfunction, likely due to this dual function. In recent experimental studies, blocking of complement lead to a reduction in pulmonary and intestinal permeability [66]. The accumulation of neutrophils in the lung was reduced by blocking the complement factor C5. This is a promising finding, which can lead to novel therapeutic probabilities.

## Tissue involvement

**Figure 4:**
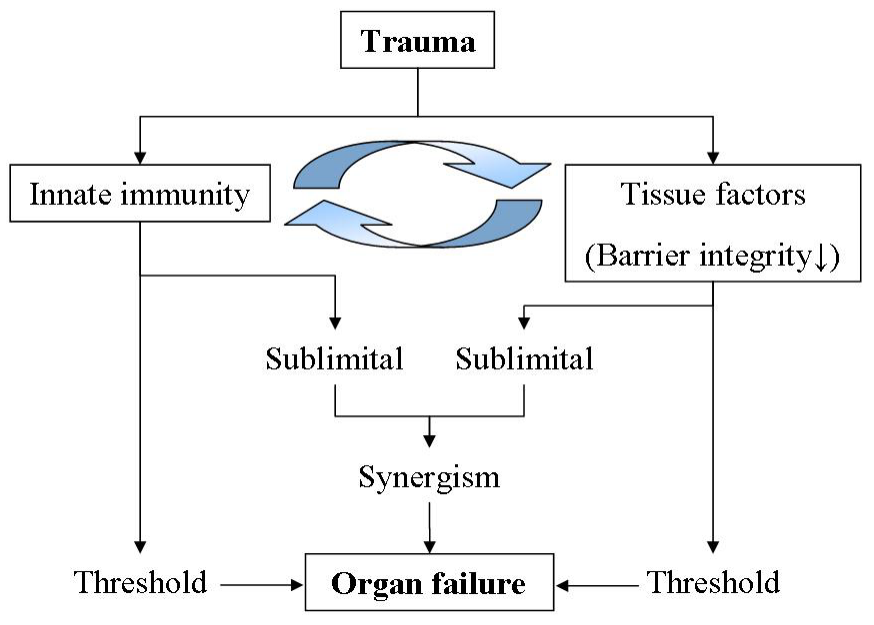
**Relation between innate immunity and tissue factors following trauma**. Shows the synergistic relation between the activation of the innate immune system and the loss of organ barrier functions. Both can act independently to promote organ failure, or when working together (synergize) induce clinical evident organ failure.

Trauma not only activates the innate immune response, but also alters the barrier integrity of several organs. Intramedullary osteosynthesis of femur fractures is thought to stimulate the innate immune response on a systemic level and is associated with an increased incidence of ARDS [67]. On the other hand, isolated thoracic injury induces local injury but is associated with the occurrence of ARDS as well [68,69]. When additional injury to the lungs is present during intramedullary osteosynthesis, the incidence of ARDS can increase two-fold [70]. This phenomenon suggests a synergistic mechanism between the activation of innate immunity and the loss of tissue barrier function (Fig. 4). The contribution of the loss of barrier function comes to attention not only in pro-inflammatory complications such as ARDS, but also in anti-inflammatory complications such as sepsis. A correlation has been shown between increased intestinal permeability and the occurrence of infectious complications [71]. It is thought that bacterial translocation due to increased intestinal permeability cause septic complications in an immunocompromised host [72]. In the pro-inflammatory phase, organ failure often precedes infection and an additional infection "only" deteriorates the remainder of the organ functions. This can be explained by the danger model, which states that innate immunity is already triggered after trauma, but can receive an additional stimulus in the form of invading bacteria. During the anti-inflammatory phase infection often precedes organ failure, giving it a more prominent role in the development of this severe complication. Despite the clear correlations between increased intestinal permeability and the incidence of sepsis in experimental settings, the relation in the clinical setting is less clear [73,74]. It is also known that the interpretation of immunological signals by cells of the innate immune system is dependent on environmental and tissue specific factors and for complications to become clinically evident, a threshold needs to be reached in specific tissues.

A cut-off point of >800 pg/ml IL-6 has been proposed as a prognostic marker and has been suggested for immunomonitoring in the damage control strategy. Unfortunately, at present no scoring system or prognostic tool is conclusive enough to adequately predict an adverse outcome on an individual level. The complexity of organ failure and the often ambiguous role of the different factors prevents a clear cut target for therapy. Many studies investigated individual mediators or effectors, which limits the interpretation of effector function in the tissues. Furthermore, cytokines often have crosstalk or cumulative effect and insight in the group effect of cytokines and chemokines would provide more accurate information about the net effect.

The scoring systems ought to be used to define the appropriate therapy. Damage control surgery and damage control orthopedics are currently used strategies to limit the incidence of organ failure after trauma [76,77]. Timing of surgery is essential in this damage control approach and recent literature provides a timeframe for planning interventions [78,79]. This timeframe, which is based on database analysis, is not fully complementary with the activation status of the innate immune system. According to the measurements of neutrophils (oxidative burst and L-selectin) hyper-inflammation is at its maximum 6 hours after trauma, whereas according to the damage control timeframe hyper-inflammation is present between day 2–4 [20,26]. Despite this problem in defining the timeframe, solutions are sought to prevent the excessive inflammation. A recent therapy that became available, hemoglobin based oxygen carriers as alternative for packed red blood cells, show promising results in limiting the inflammatory response [28]. The start of hypo-inflammation is less well defined and more individual determined, which makes therapy more difficult.

## Conclusion

Several studies have shown a relationship between the severity of trauma and the resulting immune response [75]. The injury to the host can be expressed in scoring systems and these have become important prognostic tools to calculate the risk based on clinical signs and symptoms in combination with inflammatory parameters [68]. It is likely that a threshold needs to be reached before clinical symptoms become evident. The loss of barrier integrity of different organs seems to play a major role in the development of complications in both the pro-inflammatory period and the anti-inflammatory period. Studies which focus on the interaction between host and innate immunity are to be performed to resolve the post-traumatic complications resulting in organ failure. Immunomonitoring with interpretation of group effects of cytokines or analysis of effector cells in interaction with tissue may lead to more intensive immunomonitoring and the adjustment of therapeutic and supportive strategies for the optimalization of care for trauma-patients.

## Abbreviations

ARDS: Acute respiratory distress syndrome

CARS: Compensatory anti-inflammatory response syndrome

CRP: C-Reactive protein

GM-CSF: Granulocyte macrophage colony stimulating factor

HMGB-1: High mobility group box 1

ICAM-1: Intercellular adhesion molecule 1

IL-n: Interleukin-n

ISS: Injury Severity Score

MAC-1: Macrophage 1

MARS: Mixed antagonist response syndrome

MHC-II: Major histocompatibility complex II

MIF: Macrophage migration inhibitory factor

MODS: Multiple organ dysfunction syndrome

MOF: Multiple organ failure

ROS: Radical oxygen species

sICAM: Soluble ICAM

SIRS: Systemic inflammatory response syndrome

TGF: Tumor growth factor

TNF-α: Tumor necrosis factor α

## Competing interests

The author(s) declare that they have no competing interests.

## Authors' contributions

**FH **participated in the design of the review and drafted the manuscript.

**LK **revised the manuscript critically on the content of effector processes till the final version was reached.

**GR **revised the manuscript critically on the mediator processes till the final version was reached.

**LL **participated in the design of the review and revised the manuscript till the final version was reached.

The authors have read and approved the final manuscript.
